# Murderous Assault on the Visiting Physician in the Maryboro' Lunatic Asylum

**Published:** 1859-04-01

**Authors:** 


### MURDEROUS ASSAULT ON THE VISITING PHYSICIAN IN THE MARYBORO' LUNATIC ASYLUM.

"We copy the following report of this very serious occurrence from the local paper (*The Leinster Express*); up to the moment we write it has not been followed by any alarming consequences. The nature and amount of the injury has not been overstated, but rather the contrary. Until a strict and searching inquiry be made as to the cause of this and other outrages in this Asylum, we must refrain from more particular comment:—

"An occurrence which might have produced disastrous consequences took place in this institution on last Monday. A female patient, named Margaret Kelly, complaining of a headache, was permitted to remain in her sleeping-room, where she dressed herself, putting on a cloak over her clothes; on Dr. Jacob paying her a visit, accompanied by the nurse and matron (the latter remaining outside the door), he observed that the patient, who, on his entrance, had been reclining over the bedclothes, rose slowly to a standing position on the bed without making any manifestation of anger or violence. Dr. Jacob was about retiring from the room, suspecting the woman to have some evil intent, when she drew from under her cloak a stone nearly 8lbs. weight, wrapped in a cloth, which she swung around, and struck him in the head, breaking in his hat, and inflicting a wound of nearly two inches in length. Dr. Jacob then withdrew from the room when the patient struck the nurse, causing a wound in her head also; the doctor immediately returned to the nurse's assistance, when the patient was secured. Were it not that this gentleman wore a very strong hat, the probability is that his life would have been sacrificed; happily the injury has not been so severe as to prevent him from attending his duty with caution. It has been directed by a board, at the suggestion of the inspectors, that Margaret Kelly will be brought to trial with a view to make her amenable to the criminal law; and eventually to place her in the Asylum for Criminal Lunatics at Dundrum, as she has expressed a fixed determination to take the life of Dr. Jacob and the nurse. An investigation is to take place before the next board, to ascertain how it was that the woman was allowed to be in possession of so deadly a weapon."*

At a meeting of three members of the Board of Governors since held to transact other business, it was resolved—" That in accordance with the suggestion of the inspectors, the Sessional Crown Solicitor be instructed to take legal proceedings in the case of Margaret Kelly, and that the manager do furnish him a copy of this resolution." It was also resolved—" That in consequence of the atrocious attempt made on the life of Dr. Jacob, the Board feel it to be their duty to express their determination to visit with the severest penalty in their power any neglect of an officer or nurse which might lead to the repetition of such an outrage." So far the three Governors. The clerk of the Lunatic Asylum's office, in Dublin, writes to Dr. Jacob :—" That with regard to taking means to guard against a like occurrence in future, he has to state that increased vigilance on the part of the attendants, and a rigid adherence to the Privy Council's rules, seem to the Inspectors to be the most effectual way of preventing such acts, and they have accordingly caused a communication to be addressed to the Resident Physician with that object." The Resident Physician is " the Manager" referred to in the resolution of the Governors, and the following is the communication addressed to him by the Inspectors—" They have to request that you will caution the attendants to be particular in the observance of the rules, and in examining the patients when they come in from the grounds, to ascertain if they have secreted stones or other dangerous weapons." And so the matter rests as far as the authorities are concerned; but is it so to rest? For several years the internal discipline and management of this Asylum has been the cause of complaint, and has been repeatedly brought under the consideration of the Governors, the Inspectors, the Chief and Under Secretaries at the Castle, and even of the Lord Lieutenant and Privy Council, without any adequate inquiry or examination being instituted as to the charges preferred or the irregularities alleged. The Commissioners who lately presented a report on the Lunatic Asylums of Ireland were intimately acquainted with all the particulars of these complaints and charges, yet did they refuse to enter into any inquiry as to their truth, or as to the grounds upon which such serious allegations were publicly and officially made. There is now before Parliament a bill founded on that report, and referred to a select committee of the House of Commons, and it now remains to be seen whether or not the working of the present law, as illustrated by the proceedings in this case, is to be really brought to light. A searching investigation as to this really unintelligible matter would probably afford a clue to guide political investigators of Irish affairs as to the nature and source of a certain description of influence, which is often found operating in the production of results of this description; and might, perhaps, suggest speculations in political philosophy not hitherto entertained.—*Dublin Med. Press.*

* It occurs to us that it would be farcical to institute *legal* proceedings in a case like this. If the culprit is a dangerous lunatic, let her be removed to a place of safety and security, to *Dundrum* Asylum if they please ; but it would be a mockery of justice to place her at the bar of a criminal court with the view of ascertaining her responsibility for the grave and serious offence she has committed.—EDITOR.

